# Defined Single-Gene and Multi-Gene Deletion Mutant Collections in *Salmonella enterica* sv Typhimurium

**DOI:** 10.1371/journal.pone.0099820

**Published:** 2014-07-09

**Authors:** Steffen Porwollik, Carlos A. Santiviago, Pui Cheng, Fred Long, Prerak Desai, Jennifer Fredlund, Shabarinath Srikumar, Cecilia A. Silva, Weiping Chu, Xin Chen, Rocío Canals, M. Megan Reynolds, Lydia Bogomolnaya, Christine Shields, Ping Cui, Jinbai Guo, Yi Zheng, Tiana Endicott-Yazdani, Hee-Jeong Yang, Aimee Maple, Yury Ragoza, Carlos J. Blondel, Camila Valenzuela, Helene Andrews-Polymenis, Michael McClelland

**Affiliations:** 1 Department of Microbiology and Molecular Genetics, University of California Irvine, Irvine, California, United States of America; 2 Departamento de Bioquímica y Biología Molecular, Facultad de Ciencias Químicas y Farmacéuticas, Universidad de Chile, Santiago, Chile; 3 Department of Microbial Pathogenesis and Immunology, Texas A&M University, College Station, Texas, United States of America; University of Osnabrueck, Germany

## Abstract

We constructed two collections of targeted single gene deletion (SGD) mutants and two collections of targeted multi-gene deletion (MGD) mutants in *Salmonella enterica* sv Typhimurium 14028s. The SGD mutant collections contain (1), 3517 mutants in which a single gene is replaced by a cassette containing a kanamycin resistance (Kan^R^) gene oriented in the sense direction (SGD-K), and (2), 3376 mutants with a chloramphenicol resistance gene (Cam^R^) oriented in the antisense direction (SGD-C). A combined total of 3773 individual genes were deleted across these SGD collections. The MGD collections contain mutants bearing deletions of contiguous regions of three or more genes and include (3), 198 mutants spanning 2543 genes replaced by a Kan^R^ cassette (MGD-K), and (4), 251 mutants spanning 2799 genes replaced by a Cam^R^ cassette (MGD-C). Overall, 3476 genes were deleted in at least one MGD collection. The collections with different antibiotic markers permit construction of all viable combinations of mutants in the same background. Together, the libraries allow hierarchical screening of MGDs for different phenotypic followed by screening of SGDs within the target MGD regions. The mutants of these collections are stored at BEI Resources (www.beiresources.org) and publicly available.

## Introduction

*Salmonella enterica* are found in many hosts including humans and their agricultural and companion animals. This bacterial species consists of a few known typhoidal serovars, such as *S*. Typhi or *S*. Paratyphi, that induce life-threatening enteric fever, and a large number of non-typhoidal serovars (NTS). In humans, NTS cause approximately 94 million cases of gastroenteritis and 155,000 deaths each year [1] and are the leading bacterial pathogens responsible for foodborne gastroenteritis [2], [3].

*Salmonella enterica* serovar Typhimurium ATCC14028 is a widely studied strain that was originally isolated by the Center of Disease Control in 1960 from pools of hearts and livers from 4-week-old chickens. Although many strains of serovar Typhimurium, including this strain, cause a systemic disease in BALB/c mice [4]–[6], this strain causes self-limiting gastroenteritis in virtually all hosts with an intact immune system. The strain persists in the intestine of immunologically intact mice including 129Sv and CBA/J [7] and in chicks [8]–[11]. The genome of this strain has been sequenced and annotated [12].

A widely used approach to identify a gene function is to generate a mutant with a deleted individual gene, followed by screening of the mutant for a phenotype. Generation of these mutants one at a time is time-consuming, so a resource to quickly obtain such mutants is of considerable interest to the research community. With this in mind, collections of defined deletion mutants have been established for several organisms in the past, including *Escherichia coli* K-12 [13], [14], *Acinetobacter baylyi* ADP1 [15] and several strains of the yeast *Saccharomyces cerevisiae* [16], [17]. The importance of establishing collections of such defined mutants is also recognized for metazoans such as *Arabidopsis thaliana* [18], *Caenorhabditis elegans* [19] and zebrafish [20]. These resources are invaluable for screening individual mutants for phenotypes [21], and for studies of epistasis and synthetic lethality [22]. As *Salmonella* Typhimurium is a widely used model organism, building a similar resource for *Salmonella* makes sense.

We report on the generation of the first comprehensive collections of both single-gene deletion for *Salmonella enterica* sv Typhimurium. We also present a first-of-its-kind set of systematic multi-gene deletion mutants. These collections are constructed with two markers to facilitate the subsequent combination of multiple mutations into the same genetic background. We further discuss how these resources are useful in infection models, where a high complexity of a population of different mutants is difficult to maintain throughout the entire process [23], and in situations where a host response to *Salmonella* must be screened, one mutant at a time [24].

## Results

### Generation of specific single-gene deletions in strain 14028

The GenBank annotation of *Salmonella enterica* sv Typhimurium 14028 contains 5372 protein coding sequences (CDSs) (GenBank GI:378448274). We excluded deletion targets for the following classes: rRNAs; tRNAs; ∼150 CDSs with syntenic orthologs known to be essential in three *Escherichia coli* K12 isolates [13], [14], [25], [26]; ∼100 structural elements of active lysogenic phage; most CDSs that occurred in more than one copy in the genome; and ∼750 CDSs under 100 amino acids in size that had no orthologous annotated counterpart in *S.* Typhimurium LT2 [27]. The genes not attempted for deletion are annotated with “nd” in **Table S1**.

We targeted the remaining genes including sRNAs for single-gene deletion (overall 4203 genes from strain 14028s). Primers were designed such that the resulting mutants would keep the first and last 30 bases of each targeted gene intact, to minimize interference with neighboring genes, while replacing the rest of the gene with an antibiotic resistance cassette. Primer sequences and positions can be found in **Table S1** for all experiments that succeeded in mutant generation. For 26 CDSs, the mutant was generated in more than one experiment and listed twice.

The procedure for sequence replacement with an antibiotic resistance cassette followed the Lambda-Red technique [28]. The few differences from the original plasmid are described in detail elsewhere [23]. Briefly, we added a T7 RNA polymerase promoter, positioned such that it would generate a unique transcript from the *Salmonella* genome directly downstream of each mutant, thereby creating a mutant-specific RNA “signature”.

**Figure 1:**
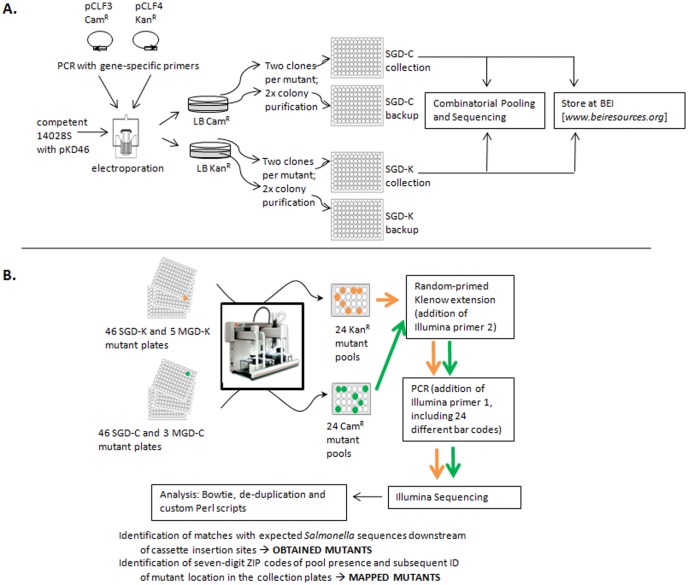
Workflow of collection construction and verification. A. General procedure and logistics of mutant generation, storage and characterization. B. Identification of obtained mutants and mapping of mutants to specific wells by a “Hypercube” Combinatorial Pooling approach, essentially as described in [30].

Each gene was deleted as both a Cam^R^ and a Kan^R^ construct in the same electroporation, and plated on separate selective plates (Figure 1A). The Kan^R^ and Cam^R^ resistance genes were oriented in the sense and anti-sense direction of the gene, respectively, to maximize any differences in polarity due to the active antibiotic resistance promoter. Deletion attempts in 4203 genes obtained at least one kanamycin-resistant (Kan^R^) or chloramphenicol-resistant (Cam^R^) mutant in 90% of cases. In most cases, two mutants of each type (two Cam^R^ and two Kan^R^) were saved separately in individual wells in 96-well plates. **Table 1** summarizes the overall results of all mutant construction attempts, and **Table S1** presents the details of each mutant. Note that we also targeted a few genes annotated in *S.* Typhimurium LT2, but not annotated in 14028s (22 CDSs and 22 sRNA candidates), and included mutagenesis attempts for a few regions in the 14028s genome where alternative annotations had suggested a CDS to be present (31 mutants). We did not include the mutants that resulted from those efforts in the summary in **Table 1**, but they are displayed in detail in **Table S1**.

**Table 1:** Summary of collection statistics.

				Targeted			Made						Mapped					
	Genomic features	Number of features	subset of “essential” features	SGD_Kan	SGD_Cm	MGD_Kan/MGD_Cm	SGD_Kan	SGD_Cm	MGD_Kan	MGD_Cm	SGD (Kan + Cm)	MGD (Kan + Cm)	SGD_Kan	SGD_Cm	MGD_Kan	MGD_Cm	SGD (Kan + Cm)	MGD (Kan + Cm)
***Genome***	CDS	5372		4094	4046	3982	3425	3279	2534	2789	3673	3461	3243	3082	2215	2632	3601	3326
			245^a^	90	88	65	21	20	40	42	23	55	21	18	32	41	23	53
	ncRNA	9		8	8	9	6	7	3	4	7	5	6	7	2	4	7	5
	rRNA	22		0	0	21	0	0	0	0	0	0	0	0	0	0	0	0
	tRNA	85		2	2	73	2	1	6	6	2	10	2	1	5	6	2	9
***Plasmid***	CDS	102		98	98	0	83	88	0	0	90	0	79	84	0	0	89	0
	ncRNA	1		1	1	0	1	1	0	0	1	0	1	1	0	0	1	0
***All***		5591		4203	4155	4085	3517	3376	2543	2799	3773	3476	3331	3175	2222	2642	3700	3340

a CDS where no transposon insertion was found in a high complexity random transposon library [29]. List of mutants present in our collections. All *S*. Typhimurium strain 14028s annotated features [12] are presented along with their status in our single-gene deletion (SGD) mutant collections. Corresponding genes in *S*. Typhimurium strain LT2 [27] that have at least 70% DNA identity and at least 70% coverage are annotated. The SGD collections are listed in genome order, followed by the multi-gene deletion (MGD) collections in genome order. For MGDs, only those primer pairs that resulted in successful deletion in at least one of the two varieties (Kan or Cam) are shown. Primer sequences, their genome locations, and the results of mutant generation, verification, and mapping to 96-well plates are shown. Empty wells are not listed.

Because both the Kan^R^ and the Cam^R^ PCR products to target each gene were transformed simultaneously, it was possible for both resistance cassettes to integrate into a single genome during the recombination event. Only nine such double mutants (5 in the SGD-K collection and 4 in the SGD-C collection) were identified by screening on LB agar plates supplemented with both Kan and Cam among over 8000 deletions. These double resistant mutants are annotated in **Table S1**.

### Generation of specific multi-gene deletions in strain 14028

To establish which regions of the genome would survive deletion when growing in rich media, we analyzed the locations of 230,000 independent EZ-Tn5 <T7/KAN-2> (Epicentre Biotechnologies) insertion sites in the genome of strain 14028 [29]. Analysis of these locations resulted in identification of regions that would not tolerate disruptions (“essential regions”). We attempted deletion of 412 intervening “non-essential” regions of which 198 MGD-K and 251 MGD-C mutants were successfully constructed. Primer pairs that resulted in a successful multi-gene deletion in at least one variety (Kan or Cam) are listed in **Table S1**. Primers flanking the MGD regions to be deleted were offset from the outer ends of the 5′ and 3′ bordering genes by 60 nucleotides, allowing clear distinction from the primers used in the SGD collections where an offset by 30 nucleotides was used.

### Identification of mutants and their locations in the library of 96-well plates

We used a combinatorial pooling approach [30] followed by Illumina sequencing to unambiguously verify the existence of mutants and simultaneously identify their plate and well locations in the collection (outlined in **Figure 1B**). Every Kan^R^ mutant (including both SGD-K and MGD-K mutants) was pipetted into a unique combination of seven out of twenty-four pools. The same procedure was performed for every Cam^R^ mutant (an additional twenty-four pools). Following a specific library preparation protocol (see **Materials and Methods**), sequence analysis then identified the presence of each mutant in each of the pools, thereby revealing the zip-code which pointed to a specific well in the collection.

During analysis of expected versus obtained zip-codes for each mutant, perfect concordance was found in the vast majority of cases. However, the robot occasionally failed to deposit a droplet into a pool or deposition of a droplet occurred during movement of the robotic arm. If over 75% of the digits of the obtained zip-codes concurred with the addresses in the expected zip code for that mutant, we assigned the mutant to that expected well. This was possible because we maximized the distance between the patterns used (error encoding), so that imperfect matches to the expected patterns still allowed mapping to a most probable well. Mutants that were present in the collection but remained un-mappable were also recorded.

This analysis identified a few systematic handling errors such as rotated plates that had occurred during collection establishment, leading to some mutants being identified in a well other than the one originally expected. These mis-assignments were corrected and the correct assignments are shown in **Table S1**.

For deletion of individual genes we attempted generation of mutants in 4203 genes of strain 14028s. Sequencing confirmed mutant generation for 3517 SGD-K and 3376 SGD-C clones, covering a total of 3773 genes in at least one variety. Analysis of the obtained zip-codes for each mutant successfully mapped 3331 SGD-K and 3175 SGD-C clones (covering a total of 3700 genes).

For multi-gene deletions, we targeted 412 regions spanning, in total, 4085 annotated features of the *S. enterica* sv Typhimurium 14028s genome. Sequencing of the junction of the mutants confirmed mutant generation for 198 MGD-K and 251 MGD-C clones, covering a total of 3476 genes. Zip-codes mapped 162 MGD-K and 234 MGD-C clones (covering a total of 3340 genes). Failed mutants may have been unfit, and smaller deletions in these regions can subsequently be attempted. Overall, the well locations of 93.8% of Kan^R^ mutants and 94.1% of Cam^R^ mutants were assigned with high probability to a single well (**Table 1**, **Table S1**).

Our sequencing approach was ideal for detection of systematic plate handling errors during assembly and storage of the collection and for detection of mutants in unexpected locations. The method was not designed to detect low levels of cross-contamination events where multiple mutants reside in the same well. A few such instances were detected through manual curation of the sequencing data. We tested 304 Kan^R^ mutants and 231 Cam^R^ mutants by PCR using primers to the flanking genomic regions of the expected mutant [23]. A cross-contaminant as well as a duplicated gene (see below) would have generated a second visible PCR product, particularly if the targeted mutant gene was less than 2 kb in size. We did not detect cross-contamination in any of these mutants. The few suspected and known contaminants are annotated in **Table S1**. Users should be aware that it is possible for additional wells to contain both an identified expected mutant and an unidentified contaminating mutant, most likely from an adjacent well.

### Identification of insertions with functional T7 promoters

We investigated whether the T7 promoter introduced during mutagenesis was functional, using a previously described procedure [23] (see also **Materials and Methods**). DNA that included the T7 promoter in the mutant and flanking *Salmonella* genome sequence was PCR amplified. The resulting DNA was used to create fluorescently labeled cRNA with T7 RNA polymerase. The cRNA was hybridized to an array consisting of about 385,000 unique oligonucleotides, representing the entire genome of strain ATCC14028 on both strands in overlapping tiles at 12 bp intervals [31]. In three biological replicates and a total of five hybridizations, we repeatedly identified labeled transcript in the correct orientation and the correct location for the 3′ end of at least 3436 Kan^R^ mutants from our SGD-K collection where an annotated 14028s gene was deleted, and from at least 3280 such expected insertions from our SGD-C collection. We interrogated the presence of functional T7 promoters in our MGD-K collection by five microarray hybridization experiments. A transcript was detected in all 198 Kan^R^ mutants in the majority of these hybridizations. The status of detection of the expected transcripts for the mutants in all collections is listed in **Table S1**. The MGD-C collection was not checked for T7 promoter activity.

### Evasion of mutagenesis by gene duplication

During establishment of the *E. coli* KEIO collection of targeted deletions in individual genes [13], [14], 25 of 7728 successfully established mutants evaded a gene knockout through duplication of the targeted gene. Most of these genes are now known to be essential or near-essential and we did not attempt to delete orthologs of this class of genes in *Salmonella*.

As mentioned above, we checked the first 304 mutants of our SGD-K collection and the first 231 mutants of our SGD-C collection by PCR using primers flanking the gene and found no evidence of duplication [23]. Next, we looked for mutants that appeared to be successfully deleted in our collection but might be near-essential, i.e. those that displayed a low frequency of transposon insertions in our previous work [29]. We investigated 30 Kan^R^ mutants and 33 Cam^R^ mutants for possible gene duplications including mutants in five genes with very few transposon insertions, indicating that these genes might be difficult to delete. We tested these candidates by qPCR amplification using primers inside the target genes (see **Materials and Methods**). In five mutants (two Kan^R^ and three Cam^R^) a duplication of the gene was present along with the mutant. Two of these genes were among those selected because they had few transposon insertions and were, thus, perhaps hard to delete. The results of this assay are included in the comments column of **Table S1**. It is likely that a few other mutants in the collection contain duplicates. In some cases it is possible that these duplications fully restore fitness.

### Collection usage alerts

Cross-contamination of some wells, as reported for the *E. coli* mutant collection [14], was also observed in our collection, and is likely to continue to occur when using the collection. It is also possible that a few successful deletion mutants may have second site mutations that alter their phenotypes, especially if these second site mutations improve growth. As is always good practice, we recommend mutant verification and transfer into a clean background for mechanistic follow-up studies.

## Discussion

We present collections of defined single-gene and multi-gene deletion mutants in *Salmonella enterica* sv Typhimurium ATCC14028. Two collections are single-gene deletion (SGD) mutants in genes not required for growth in rich liquid media (LB), marked with either kanamycin resistance (Kan^R^) or chloramphenicol resistance (Cam^R^). The two additional collections of mutants are multi-gene deletion (MGD) mutants in chromosomal regions encompassing three or more genes that are not needed for growth of *Salmonella enterica* sv Typhimurium in LB, also marked with Kan^R^ or Cam^R^. The generation of libraries with two different markers allows all viable pairwise combinations of deletions to be readily assembled in the same strain by generalized transduction.

The selectable marker within each insertion can be removed by FLP recombination leaving an in-frame scar, to diminish or eliminate polar effects on adjacent genes caused by the selective marker and its promoter [23], [28]. FLP recombination also allows three or more deletions to be assembled in the same isolate by sequential removal of antibiotic resistances followed by addition of a new marked mutation.

There are other advantages of these collections. Researchers that are engaged in functional genomics sometimes want to screen for as many phenotype-associated genes as possible in a single experiment. For this purpose, various techniques have been devised that employ pools of strains carrying random transposon mutant integrations, and monitor the change in representation of each mutant in the population [29], [30], [32]–[42]. To guarantee transposon insertions in almost all genes on the genome, including short genes, the number of random mutants that needs to be generated and screened is very high. Screening pools of large numbers of mutants is trivial in most *in vitro* conditions. However, in complex environments, such as infections in animals, the number of wild type bacterial cells surviving various stresses may be low at one or more bottlenecks, even if a huge initial dose is used.

To overcome random loss of mutants in complex environments, alternative strategies that use decreased numbers of mutants must be employed. First, one can simply reduce the number of random transposon insertion mutants in the pool used for infection [42]–[45]. This strategy lowers the number of genes screened or requires screening of separate sub-pools in different animals. Alternatively, one can use a subset of individual mapped transposon insertions, an approach that has been used in *Staphylococcus aureus* [46], *Vibrio cholerae* [47], *Pseudomonas aeruginosa* PAO1 [48], [49], and *Burkholderia thailandensis* [50]. Finally, mutants can be built with a defined deletion in each gene. Because only a single mutant is generated for each gene, the total number of different mutants required in the pool for maximum coverage of the genome is minimized.

Using defined mutants has a number of advantages: (a) Defined deletions usually involve deletion of the whole gene, instead of an insertion at an arbitrary location. (b) The mutants can be made in a way that the selectable marker and promoter can subsequently be removed in frame to downstream minimize any polar effects. (c) Different selectable markers can be introduced at the same location. Multiple markers permits construction of mutations in multiple genes in the same strain to measure, for example, redundancy or epistatic effects [14]. (d) Groups of adjacent, continuous, non-essential genes can be deleted, *en bloc*, so that a library of a few hundred mutants can encompass thousands of genes.

Large deletions are of utility in certain situations, such as screening organs after oral infection, where severe bottlenecks occur that cause random loss of mutants from complex pools. When a bottleneck allows only 20,000 bacteria to survive a step during infection, our MGD libraries of a few hundred bacteria can be used successfully. After such an MGD study identifies candidate genetic regions with functions in the investigated process, a smaller pool of only those SGD mutants representing the candidate regions can then be used to pinpoint the exact gene(s) with a function. We have used parts of the MGD and SGD libraries to validate this approach [51], [52].

The MGD collections are also useful in conditions where a host response elicited by specific genes of the bacterium is of interest. A pool of mutants cannot be used in such a scenario, because most mutants in the mixture will retain a functioning trigger and will elicit the response. Instead, such studies must be performed one mutant at a time. Part of our MGD library has been used in a hierarchical screen to identify regions in the *Salmonella* genome involved in down-modulation of expression of the human T-Cell Receptor, inhibition of blastogenesis, and proliferation of T cells, with subsequent mapping to single genes using SGDs [24].

### Collection availability

All four collections have been deposited with BEI Resources (www.beiresources.org), with the following catalog numbers: SGD-K: NRC29399–29409, NRS42824–42858, NRS42859 (partial); SGD-C: NRC29410–29420, NRS42859 (partial), NRS42865–42899; MGD-K: NRS42861–42864; MGD-C: NRS42900–42902. Note that BEI will provide a maximum of 10 mutants at a time. Researchers interested in studies of mutant pools of our collections should contact MM directly.

## Materials and Methods

### Strain

ATCC14028s, a commonly used laboratory strain, was obtained from Dr. Don Guiney (UCSD), but was originally obtained from ATCC. We re-sequenced this strain to over 10× coverage [23] and found no differences with the published sequence [12].

### Mutant generation

Primers for deletions were based on the published GenBank annotation of *S*. Typhimurium strain ATCC14028s [12] (GenBank GI:378448274) supplemented with in-house annotations using RAST and JCVI tools and the annotation of *S*. Typhimurium strain LT2, a very close relative [27]. Primers used in this project, along with their targeted coordinates in the 14028 chromosome and virulence plasmid (pSLT), mutant generation success and locations of the mutants in the collection plates are shown in **Table S1**. Wells without mutants either failed to produce a mutant or were blank.

The generation of targeted deletion mutants using the Lambda-Red mutagenesis is described in detail elsewhere [23], [28]. In brief, we amplified resistance cassettes from slightly modified pKD3 (Cam^R^) and pKD4 (Kan^R^) plasmids (named pCLF3 and pCLF4, respectively), using gene-specific primers. These primers are listed in **Table S1**. Unlike pKD3 and pKD4, plasmids pCLF3 and pCLF4 contain a T7 promoter to enable mutant-specific RNA signature production. All other features of pKD3 and pKD4, such as the FLP recombination sites, the ribosome binding site (RBS) and the start codon at the 3′ end of the mutagenic cassette for translational coupling, are retained (GenBank accession numbers EU629213 and EU629214 for pCLF3 and pCLF4, respectively). PCR products were used to transform competent *S*. Typhimurium ATCC14028 expressing the lambda Red recombinase by electroporation. Successful mutants were harvested from selective agar plates (supplemented with either 20 µg/ml Cam or 60 µg/ml Kan) after growth for 16–48 h. Two colonies were stocked for each mutant, only one of which was subsequently verified and deposited at BEI.

### Array-based analysis of presence and functionality of mutants in pools

Separate pools for the different mutant classes (SGD-K, SGD-C, MGD-K), containing all generated mutants in each class, were grown overnight in selective LB broth. Genomic DNA was isolated using the GenElute Bacterial Genomic DNA kit (Sigma). This was done in three replicates for the SGD-K and MGD-K pools and in two replicates for the SGD-C pool. The region directly 3′ from each mutation in the pools was labeled and hybridized as described in detail previously [23]. Essentially, after shearing and polyA-tailing of the genomic DNA using 40 U terminal transferase in a 50 µl reaction, nested PCR amplified the subset of polyA-tailed DNA fragments that contained the T7 promoter encoded in the cassettes integrated into each mutant, along with the flanking genomic region. In the first PCR reaction, 50 ng of purified polyA-tailed DNA was used in a PCR with 0.05 U Taq (Promega, WI) and 0.2 µM of primers FRT Out 3-1 (TTCCTATACTTTCTAGAGAA), and PolyA-P (CCT_24_VN) in 25 µl. The PCR reaction was performed under the following conditions: 94°C for 1 min; 30 cycles of 94°C for 10 s, 50°C for 10 s, and 72°C for 5 s; 72°C for 3 min. A subsequent nested PCR used 1 µl amplified product from the initial PCR in a total volume of 50 µl. Internal primer FRT Out 3-2 (TAGGAACTTCGGAATAGGAA) and primer CCT_24_VN were used under identical cycling conditions. *In vitro* transcription using the AmpliScribe T7 transcription kit (Epicentre) and labeled Cy5- or Cy3-UTP (GE Healthcare) generated cRNA that was hybridized to a 384 k Nimblegen oligo tiling array covering the entire genome of *S.* Typhimurium 14028 in 12 bp overlaps. In each hybridization, 4 µg of labeled RNA was mixed with alignment oligo, hybridization buffer and Component A according to the Roche/Nimblegen hybridization protocol, and hybridized at 42°C for 16 hours. Arrays were washed according to the manufacturer's protocol, and scanned using a GenePix 4000B laser scanner (Molecular Devices, Sunnyvale, California) at 5 µm resolution. Each replicate gDNA for each pool was hybridized at least once. Array signals were quantified and analyzed using NimbleScan v2.4, WebarrayDB [53] and custom Perl scripts. For the SGD pools, a mutant was deemed to be present with an intact T7 promoter if the median signal intensity of the five closest strand-specific probes downstream from the expected insertion site of that respective mutant was higher than the 90^th^ percentile of the intensity values of all nonsense probe intensities on the array (i.e. the probes on the array that were hybridizing to the opposite strand of the expected T7 transcripts in the collection, which should reflect background signal). For the MGD pool, a plot of signal intensities for probes along the genome was visually inspected and all expected T7 transcript sites scored manually.

### Identification of the location of all mutants in the collections using combinatorial pooling

Following a combinatorial pooling approach [30], every Kan^R^ mutant (including both SGD-K and MGD-K mutants) was initially pipetted robotically into a unique combination of seven out of twenty-four pools. The same procedure was performed for every Cam^R^ mutant. During this pipetting step, 24 pools of Kan^R^ mutants (P01–P24) and 24 pools of Cam^R^ mutants (P25–P48) were generated that each contained between 1325 and 1496 individual mutants, with every mutant being deposited in 7 different pools. A custom Perl script identified sufficient unique 7-out-of-24 pool combinations that each varied from all others by at least two pools. The resulting well-specific seven-pool “zip code” therefore differed from any other code by at least two numbers (error encoding). As an example, the Kan^R^ mutant of gene STM14_0001, which resides in plate SGD_067/068_Kan, well G11, was deposited in pools P01, P04, P06, P10, P17, P23 and P24, and is therefore assigned the corresponding “zip code” of 01-04-06-10-17-23-24. No other mutant was deposited in a pattern that contained more than 5 of these seven pools.

Genomic DNA of the resulting 24 Kan^R^ mutant pools and the 24 Cam^R^ mutant pools was prepared using the GenElute Bacterial Genomic DNA kit (Sigma). These gDNAs were subsequently denatured for 5 min at 95°C, randomly primed using 0.2 uM Random_Multiplexing_Primer (5′- GTGACTGGAGTTCAGACGTGTGCTCTTCCGATCTNNNNNNNNN) and extended, using 50 U exo- Klenow enzyme (New England Biolabs) and a gradual increase of temperature over 6 min to 37°C, where the reaction proceeded for 30 min. Following enzyme deactivation at 75°C for 20 min and subsequent QIAquick purification (Qiagen), the 3′ ends of products were tractably amplified by PCR using 2.5 U Taq enzyme (Invitrogen) and primers Read2_Illumina (5′ GTGACTGGAGTTCAGACGTGTGCTCTTCCGATCT) and SGD_Index (5′ACACTCTTTCCCTACACGACGCTCTTCCGATCT(X_7_)GACCTAAGGAGGATATTCA, where X_7_ indicates a pool-specific known “barcode” sequence). The 5′ ends were amplified using primers Read2_Illumina and SGD_5′end_code_2 (5′ ACACTCTTTCCCTACACGACGCTCTTCCGATCTAGGAGTCTCGAAGCAGCTCCAGCCTG). In both reactions, primer Read2_Illumina was added after an initial extension cycle, following this regimen: 1 cycle of 94°C 3 min - 56°C 3 min - 72°C 5 min; addition of Read2_Illumina; 25 cycles of 94°C 15 sec - 60°C 30 sec - 72°C 1 min; final extension at 72°C 5 min. As a result of this approach, the 3′ends of the DNAs in every pool were now tagged with a specific “barcode”. Products were QIAquick purified (Qiagen) and assembled at roughly equimolar amounts.

The mixtures were then paired-end sequenced on the Genome Analyzer IIx (Illumina), and sequences were analyzed following a pipeline that involved Bowtie [54], de-duplication and custom Perl scripts to improve alignments to the genome of *S.* Typhimurium 14028. Reads that aligned within a 10-base window to expected genome locations were deemed to have been generated from that expected mutant. The “barcode” reads obtained for each mutant were then used to identify the exact physical plate/well location of each mutant in the collection. Data interpretation is reported in **Table S1**.

### Test for target gene duplication

Primers were designed to amplify 140–170 bp internal fragments of the targeted genes. A qPCR assay was then performed, in duplicate, on boiled material from each mutant well, using Evagreen dye (Biotium) and the Kapa 2G Robust Hotstart ReadyMix PCR kit (Kapa Biosystems). For each mutant, a control reaction was included using an internal primer pair for a gene that had been targeted in a different mutant. The obtained C_T_s of control PCR versus internal PCR were compared, and a mean ΔΔC_T_s above 3 indicated lack of duplication, successful mutant generation, and a lack of significant contamination with another mutant. Mutants where the difference between the C_T_s was lower than 3 were purified to single colony and tested further by PCR of the mutant junction in the genome, performed with primer SGD_5_prime_out (5′-TCGAAGCAGCTCCAGCCTG), facing outward towards the 5′ end of the resistance cassette, and a primer adjacent to each targeted gene, facing towards the 5′ end of the resistance cassette. Successful PCR amplification indicated accurate integration of the resistance cassette into the target gene. Internal PCR amplification was repeated in this sample to reveal if there was target gene duplication.

## Supporting Information

Table S1 **List of mutants present in our collections.**
(XLSX)
